# Primary Nucleation of Polymorphic α-Synuclein Dimers Depends on Copper Concentrations and Definite Copper-Binding Site

**DOI:** 10.3390/biom14060627

**Published:** 2024-05-26

**Authors:** Carmia Blacher, Karina Abramov-Harpaz, Yifat Miller

**Affiliations:** 1 Department of Chemistry, Ben-Gurion University of the Negev, Beér-Sheva 8410501, Israel; 2 Ilse Katz Institute for Nanoscale Science and Technology, Ben-Gurion University of the Negev, Beér-Sheva 8410501, Israel; 3 The School of Brain Sciences and Cognition, Ben-Gurion University of the Negev, Beér-Sheva 8410501, Israel

**Keywords:** Parkinson’s disease, metal ions, α-synuclein, amyloids, self-assembly, neurodegenerative diseases, protein aggregation, oligomers, polymorphism

## Abstract

The primary nucleation process of α-synuclein (AS) that forms toxic oligomeric species is the early stage of the pathological cause of Parkinson’s disease. It is well-known that copper influences this primary nucleation process. While significant efforts have been made to solve the structures of polymorphic AS fibrils, the structures of AS oligomers and the copper-bound AS oligomers at the molecular level and the effect of copper concentrations on the primary nucleation are elusive. Here, we propose and demonstrate new molecular mechanism pathways of primary nucleation of AS that are tuned by distinct copper concentrations and by a specific copper-binding site. We present the polymorphic AS dimers bound to different copper-binding sites at the atomic resolution in high- and low-copper concentrations, using extensive molecular dynamics simulations. Our results show the complexity of the primary nucleation pathways that rely on the copper concentrations and the copper binding site. From a broader perspective, our study proposes a new strategy to control the primary nucleation of other toxic amyloid oligomers in other neurodegenerative diseases.

## 1. Introduction

Parkinson’s disease (PD) is a neurodegenerative disease by which the pathological hallmarks are Lewy bodies and Lewy neurites that mainly comprise α-synuclein (AS) aggregates, e.g., oligomers and fibrils [1,2]. In the last two decades, it has been reported that AS oligomers are more neurotoxic to neuronal cells than fibrils [3]. Specifically, it is known that the early-stage oligomers play a vital role in the primary nucleation of the AS aggregation process [4]. Recently, the structural properties of polymorphic AS dimers at the atomic resolution have been reported using computational tools [5].

In PD brains, high levels of copper had been found in the cerebrospinal fluid (CSF) [6], and in the blood serum of PD patients [7]. AS monomers and aggregates also appear as biomarkers in the CSF of PD brains [8]. It has been shown that AS and copper were also found in the synapses and they both participate in the synaptic functions [9,10]. In fact, copper has a synergic effect on AS aggregation [9,11]. At physiological copper concentrations, it has been shown that copper accelerates the formation of AS fibrils, while not altering the fibrils’ morphology [12,13]. While early studies argue that copper accelerates AS aggregation, some caution should be considered when focusing on the molecular mechanisms by which copper binds to polymorphic early-stage oligomers. Moreover, previous studies claim that high copper concentrations increase the risk of developing PD, due to the increased AS aggregation process [14,15]. Nevertheless, this correlation is controversial and far from being clarified. Other previous studies support the theory that low copper concentrations enhance the risk of developing PD [16,17].

The copper-binding sites and their effect on AS aggregation are crucial topics in the study of molecular mechanisms of AS aggregation. Three copper-binding sites in the AS monomer have been proposed by experimental studies [18]: (1) N-terminal domain: Met1, Asp2, and Met5; (2) His50; and (3) C-terminal domain: Asp119, Asp121, Asn122, and Glu123. The copper-binding sites in AS fibrils have been investigated by computational and experimental studies [19]. It has been reported that the copper-binding site at the N-terminus demonstrated a high affinity [19,20]. It has been shown by in vitro studies that the binding affinity of this binding site is in the range of μM to nM of Cu^2+^ [21]. However acetylation at this domain of AS drastically reduces its affinity for copper binding [20,22] and has no effect on AS fibril formation [20,23]. At physiologically relevant copper concentrations, copper binds to the N-terminus at a high affinity and this supports the hypothesis that copper is associated with PD [12]. It has been suggested that the C-terminal domain is a weak copper-binding site, i.e., lower-affinity binding motif, and it enhances AS fibrillation [24,25]. Finally, it has been reported by experimental and computational studies that divalent copper ions do not bind to His50 in AS fibrils, and, thus, has no effect on fibrillation [19].

Like other amyloids, AS also presents polymorphic fibrils [26,27,28]. Hence, distinct copper binding sites increase the polymorphism in amyloid fibrils [29,30], among them AS fibrils. Obviously, polymorphic AS fibrils are derived from polymorphic AS oligomers; thus, it is expected that distinct copper-binding sites also increase polymorphic AS oligomers. Indeed, recently, it has been reported that AS oligomers are polymorphic [31,32]. To date, the copper-binding sites in polymorphic AS oligomers have not been investigated either by experimental or by computational methods.

Recently, in vitro studies showed that copper could bind to pre-formed and formed AS fibrils and affect the morphologies [33]. Yet, the effect of copper on the early-stage oligomers and morphologies at the atomic resolution are still unknown. Herein, we present the first study that investigates the effect of copper concentrations and the distinct three copper-binding sites on the primary nucleation of polymorphic AS dimers. The current work provides insights into the molecular mechanisms of the early-stage aggregation process that yield to toxic species in PD.

## 2. Materials and Methods

### 2.1. Constructions of Cu^2+^-Bound AS_1–140_ Fibril-Like Dimer Models

Amyloids present polymorphic aggregates; hence, AS fibrils are polymorphic and AS oligomers are polymorphic. Herein, we investigated four polymorphic fibril-like AS dimers that were chosen to construct a total of 24 Cu^2+^-bound AS_1–140_ dimer models: (1) model A, which was predicted by computational methods and based on experimental studies [34], (2) model B, that was solved by ssNMR (PDB ID code: 2N0A) [35], (3) model C, which is a rod polymorph that was solved by cryo-EM (PDB ID code: 6CU7) [36], and (4) model D, which is a twister polymorph that was also solved by cryo-EM (PDB ID code: 6CU8) [36]. While the full-length AS_1–140_ for model A was solved by computational tools, and model B was solved by ssNMR, the two latter models lack the N- and C-termini domains of AS, because of their disordered structures. Model C illustrates the sequence of AS_38–97_, whereas model D illustrates AS_43–83_ residues. Hence, elongation of the N- and C-termini of models C and D was performed to produce the full-length AS_1–140_ [5]. The elongation was guided by the formation of the maximum hydrophobic and electrostatic interactions between the residues along the sequence within the inter-sheets for each model.

In this work, the 24 constructed Cu^2+^-bound AS dimer complexes are distinct by the Cu^2+^ ion concentrations and by the specific Cu^2+^-binding sites. Copper concentrations were defined by the Cu^2+^:AS ratio within each AS dimer. The Cu^2+^:AS ratio of 1:1 was defined as high copper concentration and 1:2 as low copper concentration. The three intermolecular-experimental-based Cu^2+^-binding sites in the AS monomer [12,37,38,39,40] that were examined in each polymorphic AS dimer are as follows: (1) the N-terminal domain, involving Met1, Asp2, and Met5; (2) the His50 site, which is located near the NAC domain; and (3) the C-terminal domain, including Asp119, Glu121, Asn122, and Glu123. The Cu^2+^ was parametrized using experimental parameters and integrated into the NAMD software (Theoretical and Computational Biophysics Group, Beckman Institute for Advanced Science and Technology, University of Illinois Urbana-Champaign, IL, USA. Version 2.14, Release 2022). Notably, water molecules can participate in metal co-ordination to complete the geometry at each copper-binding site.

Each Cu^2+^-bound AS dimer model was constructed by applying the BIOVIA Discovery Studio Visualizer software (BIOVIA, Dassault Systèmes, BIOVIA Discovery Studio, San Diego, CA, USA. Version v21, Release 2020) and using the CHARMM force-field [41]. Figures S1–S4 illustrate the initial Cu^2+^-bound AS dimer models. Energy minimization was performed using the adopted basis Newton–Raphson (ABNR) method. Subsequently, molecular dynamics (MD) simulations were performed for each model.

### 2.2. Molecular Dynamics (MD) Simulations Protocol

The MD simulations of the solvated constructed models were performed in the NPT (N—number of particles; P—pressure; T—temperature) ensemble using the nanoscale molecular dynamics (NAMD) package, with the CHARMM36 force-field with the CMAP correlation [41]. Each Cu^2+^-bound AS dimer complex model was energy-minimized and explicitly solvated in a TIP3P water box [42,43]. Each water molecule within 2.5 Å of the models was removed. Counter ions (NaCl) were added at random locations to neutralize the charge of the models. The Langevin piston method [41,44,45] with a decay period of 100 fs and a damping time of 50 fs was used to maintain a constant pressure of 1 atm. A temperature of 330 K was controlled using a Langevin thermostat with a damping coefficient of 10 ps [41]. The short-range van der Waals (VDW) interactions were calculated using the switching function, with a twin range cutoff of 10.0 and 12.0 Å. Long-range electrostatic interactions were calculated using the particle mesh Ewald method with a cutoff of 12.0 Å [46,47]. The equations of motion were integrated using the leapfrog integrator with a step of 1 fs. Counter ions and water molecules were allowed to move. Hydrogen atoms were constrained to equilibrium bonds using the SHAKE algorithm [48]. The minimized solvated systems were energy-minimized for 2000 additional conjugate gradient steps and 20,000 heating steps at 250 K, with all atoms allowed to move. The system was then heated from 250 K to 330 K for 300 ps and equilibrated at 330 K for 300 ps. The choice of a temperature higher than the physiological temperature was carried out in order to investigate the stability of the constructed models. Structural models that are stable at high temperatures are also stable at physiological temperatures. Simulations were performed for 200 ns for each variant model. To approve the timescales of the simulations, root-mean-square deviation (RMSD) calculations were performed along the MD simulations. Overall, the simulated models exhibited convergence at the end of approximately 50 ns. Therefore, the timescale of the MD simulations in the current work justifies the choice of timescales. The structures were saved every 10 ps for analysis.

### 2.3. Structural Analyses

Metal-bound peptide structures have been explored by several structural analyses. Conformational changes in the peptide structure were obtained by conducting a root-mean-square deviation (RMSD) analysis. This allowed us to identify the convergence of each AS monomer within the dimer along the MD simulations. To evaluate the fluctuations of each residue within the AS, root-mean-square fluctuation (RMSF) analysis was performed. To compare the secondary structures of the distinct AS dimers, the database of the secondary structure of proteins (DSSP) method [49] was applied. The values of DSSP were calculated along the total time of the simulation for each model. The DSSP method provides the percentage of α-helices or β-strands located along the AS sequence.

The inter- and intra-peptide interactions were measured based on the distance between specific atoms of the residues within the AS dimers during the simulations. For hydrophobic interactions, the distance between the C_α_ atoms of two hydrophobic residues was measured using a cutoff distance of 10 Å [50]. The hydrophobic contact maps between AS monomers were described by the occurrence of interactions in the MD simulations. Finally, the hydrogen bonds between the residues within the AS monomers were measured along the MD simulations. The cutoff distance for hydrogen bonds was set to 2.4 Å [51].

Water solvation analysis was calculated separately for each AS peptide monomer, by which the number of water molecules within 3.0 Å of each residue was measured and normalized to percentages, according to the maximum number of water molecules for all models.

### 2.4. Determining the Conformational Energies and Populations for the Simulated Cu^2+^-Bound AS Dimers

For each group of models that differed by the number of atoms, that is, by the Cu^2+^: AS ratio, separate conformational energy and population analyses were calculated. First, to obtain the relative conformational energies of the Cu^2+^-AS dimer in various conformations, the trajectories of the last 5 ns were extracted from the explicit MD simulation, excluding water molecules. The solvation energies of all systems were calculated using the Generalized Born Method with Molecular Volume (GBMV) [52,53]. The hydrophobic solvent-accessible surface area (SASA) term factor was set to 0.00592 kcal∙mol^−1^∙Å^−2^ [54]. Each variant was minimized for 1000 cycles, and the conformation energy was evaluated using a grid-based GBMV. Minimization does not change the conformations of each variant, but only relaxes the local geometries due to the thermal fluctuation that occurred during the MD simulations. The 500 conformations, derived from the last 5 ns, for each of the examined models were used to construct the free-energy landscape of the conformers and to evaluate the conformer probabilities using Monte Carlo (MC) simulations. First, one conformation of conformer i and one conformation of conformer j were randomly selected. The Boltzmann factor was computed as e^−(Ej−Ei)/kT^, where E_i_ and E_j_ are the conformational energies evaluated using GBMV calculations for conformations i and j, respectively, k is the Boltzmann constant and T is the absolute temperature (298 K used here). If the value of the Boltzmann factor is larger than a random number, then the move from conformation i to conformation j is allowed. After 1 million steps, the conformations ‘visited’ for each conformer were counted.

Finally, the relative probability of model n was evaluated as P_n_ = N_n_/N_total_, where P_n_ is the population of model n, N_n_ is the total number of conformations visited by model n, and N_total_ is the total number of steps. The advantages of using MC simulations to estimate the conformer probability lie in their good numerical stability and ability to control the transition probabilities among several conformers.

## 3. Results and Discussion

**Figure 1:**
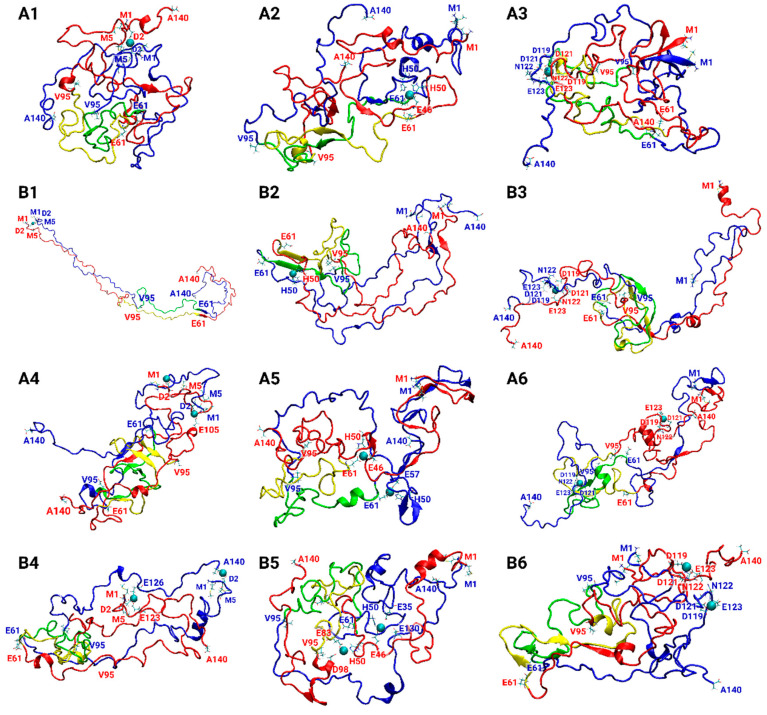
Final simulated Cu^2+^-AS_1–140_ dimer models at low copper concentrations: A1–A3, and B1–B3; and at high copper concentrations: A4–A6, and B4–B6. Descriptions of the models are detailed in Table 1.

**Figure 2:**
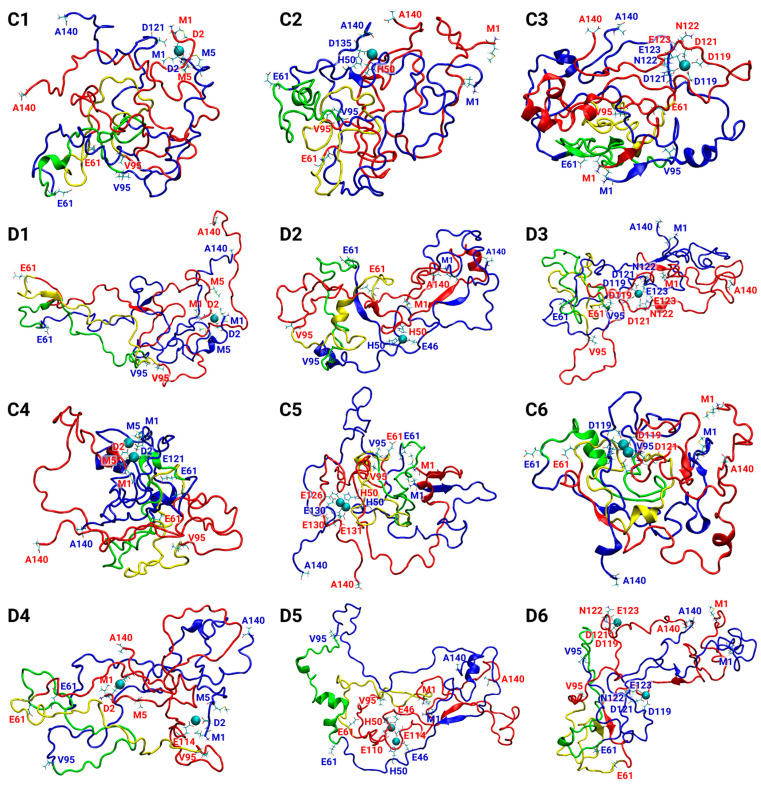
Final simulated Cu^2+^-AS_1–140_ dimer models at low copper concentrations: C1–C3, and D1–D3; and at high copper concentrations: C4–C6, and D4–D6. Descriptions of the models are detailed in Table 1.

Copper ions play crucial roles in AS aggregation, but their effect on AS aggregation is controversial. Some studies claim that copper ions promote AS aggregation [55,56,57,58,59,60,61], and, recently, it has been declared that copper ions can either accelerate or inhibit AS aggregation [18]. Moreover, it has been reviewed that the AS aggregation rate depends on the copper concentrations [61]. To resolve this controversy, we explored polymorphic AS dimers with distinct copper-binding sites at two concentrations: low and high concentrations. The low copper concentrations are defined by a sub-stoichiometric Cu^2+^:AS ratio of 1:2, and the high concentrations by a stoichiometric Cu^2+^:AS ratio of 1:1. A total of 24 models of Cu^2+^-AS dimers were separately investigated by 24 separate molecular dynamics (MD) simulations runs (using a total of 4800 ns). Table 1 illustrates the 24 polymorphic AS dimers. The initially constructed 24 models that are described in Figures S1–S4 are derived from the A, B, C, and D fibrillary models, that were solved by experimental techniques and computational tools [34,35,36,62]. The constructed models, MD simulations protocol, and analyses are detailed in the “Materials and Methods” section. The convergence of the simulated Cu^2+^-bound AS dimer models indicates that the timescales of the simulations are reasonable for all studied models (Figures S5–S8). The number of hydrogen bonds of the initial fibrillary morphologies of all AS dimer models decreased along the MD simulations (Figure S9). The fibrillary morphologies of AS present hydrogen bonds along the fibril axis, thus contributing to the cross-β structure. In the dimeric forms, these hydrogen bonds are presented between two monomers. During the MD simulations, the number of these hydrogen bonds are decreased, and, therefore, the original cross-β structure is ruined. Interestingly, all the Cu^2+^-bound AS dimer models exhibited similar solvation values of the residues along the AS sequence (Figure S10). The twelve final simulated models A1–A6 and B1–B6 are represented in Figure 1, and the twelve final simulated models C1–C6 and D1–D6 are illustrated in Figure 2.

### 3.1. Copper Concentrations Affect the Metal-Binding Sites

Three copper-binding sites in the AS monomer that were proposed by experimental studies and reviewed elsewhere [12,37,38,39,40] had been applied to construct the initial 24 Cu^2+^-bound AS dimer models: (1) N-terminal domain: Met1, Asp2, and Met5; (2) His50; and (3) C-terminal domain: Asp119, Asp121, Asn122, and Glu123. Four AS dimers were constructed based on the four polymorphic AS fibrils. For each of the four AS dimers, these three copper-binding sites had been examined at low copper concentrations (Cu^2+^:AS ratio of 1:2) and high concentrations (Cu^2+^:AS ratio of 1:1). Table 1 illustrates the 24 Cu^2+^-bound AS dimer models. The final simulated models are presented in Figure 1 and Figure 2.

**Figure 3:**
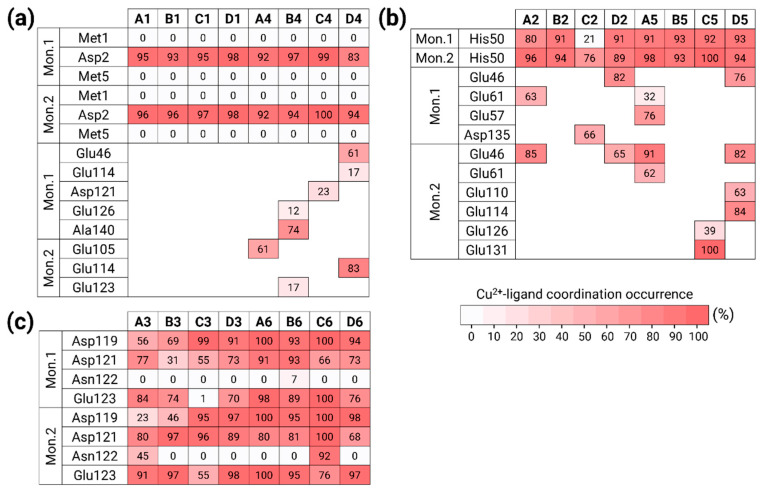
The percentages by which Cu^2+^ ions bind to each residue along the MD simulations, for the binding sites. (**a**) Met1, Asp2, and Met5; (**b**) His50; and (**c**) Asp119, Asp121, Asn122, and Glu123.

In the constructed models A1, B1, C1, and D1, the copper ion was bound to residues Met1, Asp2, and Met5 in each one of the two monomers within at low copper concentrations. Figure 3a illustrates the copper-binding sites in these models. The copper ion conserved the binding site only with Asp2 of the two monomers along the MD simulations. In our previous work, also in the forms of two polymorphic AS fibrils, copper ion was bound during the MD simulations only with Asp2 [19].

In the constructed models A4, B4, C4, and D4, the copper ions were bound to residues Met1, Asp2, and Met5 in each monomer within the dimer at high concentrations. Figure 3a illustrates the copper-binding sites of these models. Moreover, at high concentrations, the copper ions were strongly bound to Asp2 in both monomers during the MD simulations. In addition to Asp2, residues at the C-terminus participated in the copper co-ordination, including Glu residues and the carboxylic group ends of Ala140. The AS fibrillary forms also illustrated a similar scenario, by which copper ions were also bound to Glu residues at the C-terminus (and, in a few cases, also to Asp20) [19].

In the constructed models A2, B2, C2, and D2, the copper ion was bound to His50 in each one of the two monomers at low copper concentrations. Figure 3b illustrates the copper-binding sites of these models. In models A2, B2, and D2, the copper ions strongly conserved the binding site. In model C2, His50 slightly conserved the binding site, at least with one monomer, probably due to the repulsion between the two monomers in the proximity domain of His50. While, in model B2, only His50 of the two monomers bound the copper ion, in models A2, C2, and D2, further residues completed the co-ordination mode. While, in models A2 and D2, the Glu residues at the N-terminus (Glu46, Glu61) completed the co-ordination mode, in model C2, the residue Asp135 at the C-terminus completed the co-ordination mode.

In the constructed models A5, B5, C5, and D5, the copper ion was bound to His50 in the two monomers within the AS dimer at high copper concentrations. Figure 3b illustrates the copper-binding sites in these models. At high concentrations of copper, His50 in both monomers strongly binds the copper ions. While, in model B5, only His50 residues bound the copper ions, in the other models, Glu residues at the N- or/and C-termini completed the co-ordination mode. Interestingly, both models B2 and B5 are based on the same AS polymorph model B, bound only to His50. Therefore, both at high and low copper concentrations, among all polymorphic AS dimers, only the model B dimers co-ordinated to His50, and not to further residues.

It is well-documented and reviewed that copper ions can bind to His50 in the AS monomer [18], but not to AS fibrils [19]. Herein, we provide a first study that shows that His50 residues in AS dimers bind copper ions. It is known that AS fibrils are well-ordered with the cross-β structure in the proximity domain of His50; therefore, copper ions are less favored to bind to this domain. However, monomers and dimers are disordered and more flexible in the His50 domain, thus favoring the binding of copper ions.

Finally, in the constructed models A3, B3, C3, and D3, the copper ion was bound to residues Asp119, Asp121, Asn122, and Glu123 in the two monomers within the AS dimer at low copper concentrations. Figure 3c illustrates the copper-binding sites of these models. At low copper concentrations, the Asn122 residues in both monomers do not participate in the copper binding, excluding model A3, which presents Asn122 in one monomer that slightly binds copper ion. In model C3, Glu123 did not bind the copper ion in one monomer.

In the constructed models A6, B6, C6, and D6, the copper ions were bound to residues Asp119, Asp121, Asn122, and Glu123 in the two monomers within the AS dimer at high concentrations. Figure 3c illustrates the copper-binding sites of these models. At high concentrations, the Asn122 residues in both monomers do not bind the copper in the binding site except for model C6 which illustrates that Asn122 binds the copper ion strongly in one of the monomers.

Overall, some of the residues bound less to the copper ions in the monomers (less than 70%). The other residues strongly bound to the copper ions (more than 70%). In some cases, more than four residues bind to copper ions (Figure 3), although it is known that the copper ion co-ordination mode is either four or six. The metal co-ordination mode was defined by counting the percentage of residues that bound copper ions in the MD simulations. Our simulations show that there is competition between residues that bind the copper ions, but, at a particular snapshot, each copper ion binds only four atoms. Nevertheless, in all co-ordination modes of copper that were studied here, water molecules completed the co-ordination mode to four or six, e.g., in the case where His50 residues bind to copper. Notably, at high copper concentrations, all the AS dimer polymorphs (models A4–A6, B4–B6, C4–C6, and D4–D6) exhibited a higher binding occurrence in comparison to each of the corresponding lower concentrations of AS dimers. Moreover, these AS dimers occupied a higher number of additional residues that participate in the copper binding.

### 3.2. Copper-Binding Sites at Different Concentrations Affect the Primary Nucleation of AS Aggregation

**Figure 4:**
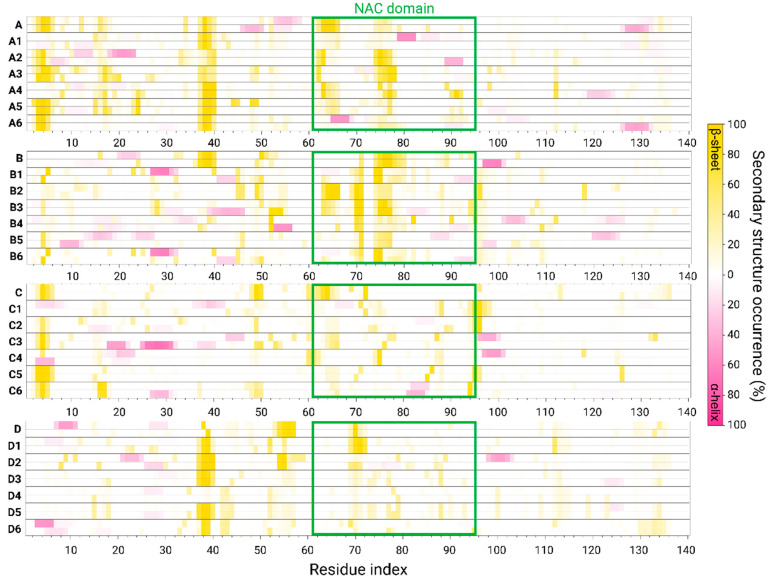
The percentages of the secondary structure along the sequence of AS in Cu^2+^-free polymorphic AS_1–140_ dimers (models A, B, C, and D) [5] and in Cu^2+^-bound polymorphic AS_1–140_ dimers (models A1–A6, B1–B6, C1–C6, and D1–D6). The sequence of the NAC domain is indicated in green rectangles.

In amyloid aggregation, primary nucleation is an important initial step in the process of fibrillation. The primary nucleation triggers the formation of β-sheets along the sequence of the amyloid and, hence, leads to the elongation of the β-sheets that eventually yields to the formation of fibrillary species. To investigate the primary nucleation in AS dimers, the secondary structures of the monomers within the dimers were computed. Figure 4 illustrates the secondary structures of the AS dimers that were investigated for the Cu^2+^-bound AS dimers and the AS dimers in the absence of copper ions, that were previously investigated by our group [5]. It has been shown that, in all polymorphic AS dimers in the absence of copper ions, the primary nucleation occurs at the N-terminus and the NAC domain [5]. Herein, the effect of the copper-binding site on the primary nucleation for each AS dimer polymorph at each copper ion concentration has been investigated.

In the case of the copper ions bound at the N-terminal domain, both at low and high concentrations for models B and C (i.e., models B1, B4, C1, and C4; see Table 1, Figure 1 and Figure 2), the copper ions disrupted the β-sheets at the N-terminus and at the NAC domain. Therefore, copper ions inhibit the primary nucleation at the N-terminal and NAC domains in these polymorphic AS dimers. In model A, at both high and low copper concentrations (i.e., models A1 and A4; see Table 1, Figure 1), there is a disruption of the β-sheets in one N-terminal domain, while the β-sheets in the other N-terminal domain were conserved. Hence, the copper ions partially inhibit the primary nucleation at the N-termini domains in this polymorphic AS dimer. In model A, at both high and low copper concentrations (i.e., A1 and A4; see Figure 1), the copper ions disrupt the β-sheets in the NAC domain, thus inhibiting the primary nucleation of the NAC domain. In model D, at low copper concentrations of copper ions (i.e., model D1; see Table 1 and Figure 2), in one N-terminal domain, there is a disruption of the β-sheets; however, in other models, in the N-terminal, there is a short β-sheet segment that may lead to primary nucleation. Moreover, while in the absence of copper ions, there is a lack of β-sheets in the NAC domain in this polymorph dimer, in the presence of low copper concentrations of copper, short β-sheets in each monomer are presented. Therefore, low copper concentrations of copper ions promote primary nucleation in the NAC domain. In model D, at high concentrations of copper ions (i.e., model D4; see Table 1 and Figure 2), there was a disruption of the β-sheets, indicating that, at high concentrations, the copper ions inhibit the primary nucleation at the N-termini. In summary, in the cases where copper ions bind to the N-terminus, the inhibition or acceleration of the primary nucleation in the NAC domain depends on the polymorph dimer and the copper concentrations.

In the case where the copper ions bound to His50, both at low and high copper ion concentrations, the β-sheets were not disrupted at the N-termini domains in models A and C (i.e., models A2, A5, C2, and C5; see Table 1, Figure 1 and Figure 2). However, the β-sheets were disrupted in the NAC domain in these polymorph dimers both at low and high concentrations. In model A2, a short β-sheet segment far from His50 in the NAC domain was formed. In model B, both at low and high concentrations (i.e., B2 and B5; see Table 1 and Figure 1), copper ions disrupted the β-sheets in both the N-termini and NAC domains. Finally, in model D, at both low and high concentrations (i.e., D2 and D5; see Table 1 and Figure 2), copper ions promoted the formation of β-sheets at one of the N-terminal domains and disrupted β-sheets in the N-terminus and the NAC domain of other models.

In summary, in the case where copper ions bind to His50 both at low and high concentrations, there is an inhibition of the primary nucleation in the NAC in the proximity domain of His50 in all polymorphic AS dimers. This observation is not surprising, because it is known that His50 is a part of the cross-β structure of AS fibrils [19,34]. Hence, the copper ions interfere with the native cross-β structure at the early stage of the aggregation process of the AS dimers’ formation.

In cases where the copper ions were bound at the C-terminal domain, at both low and high copper concentrations, the β-sheets were not disrupted in the N-terminal domains in models A and C (i.e., models A3, A6, C3, and C6; see Table 1, Figure 1 and Figure 2). However, β-sheets were disrupted in the NAC domain in these polymorph dimers at both low and high copper concentrations. Yet, in model A3, a short β-sheet segment was formed in the NAC domain. In model B, both at low and high concentrations (i.e., B3 and B6; see Table 1 and Figure 1), the β-sheets were disrupted at the N-termini and slightly shortened and disrupted in the NAC domain. In model D, both at low and high concentrations (i.e., D3 and D6; see Table 1 and Figure 2), copper ions induced the formation of β-sheets at the N-terminal domains and disrupted β-sheets in the NAC domain. In summary, typically, in cases where copper ions bind to the C-termini of polymorphic AS dimers, there is a disruption in the NAC domain, thus leading to the inhibition of the primary nucleation.

### 3.3. The Preference of Copper-Binding Site at Low Copper Concentrations Depends on Hydrophobic Contacts between the NAC Domains

**Figure 5:**
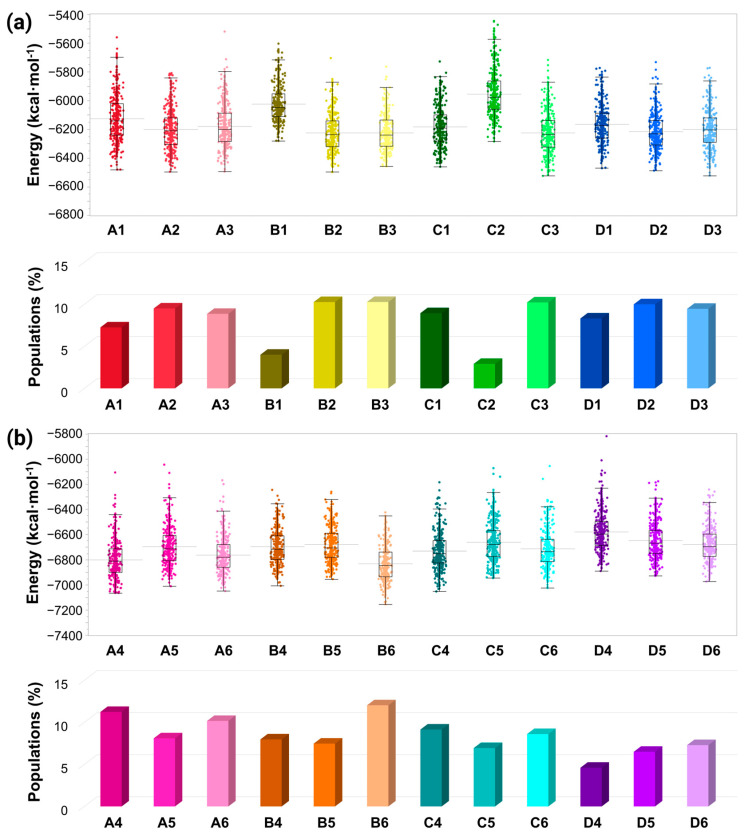
(**a**) Conformational energies (Top) and populations (Bottom) of the Cu^2+^-bound AS_1–140_ dimer models at low copper concentrations, and (**b**) conformational energies (Top) and populations (Bottom) of the Cu^2+^-bound AS_1–140_ dimer models at high copper concentrations.

Three copper-binding sites were proposed in the AS monomer [18], and had been examined in polymorphic AS fibrils [19]. While, in AS fibrils, copper ions do not bind to His50, herein, in polymorphic AS dimers, all three copper-binding sites are possible. Nevertheless, among all these three copper-binding sites, which one is preferred at low copper concentrations? To answer this question, we computed the conformational energy and populations of all the polymorphic copper-bound AS dimers at different binding sites at low copper concentrations (Figure 5a).

All polymorphic AS dimers in which copper ions bind to the C-terminal domain (models A3, B3, C3, and D3) illustrate a low conformational energy and high populations, and, thus, are more preferred. In addition, the polymorphic dimers, models A2, B2, and D2, by which the copper ions bind to His50, also demonstrated a lower conformational energy and higher populations. In the AS dimer polymorph, which is based on the experimental C model [36], the copper-binding site in His50 is not preferred; instead, the copper ions prefer to bind to the N- and C-termini (models C1 and C3).

**Figure 6:**
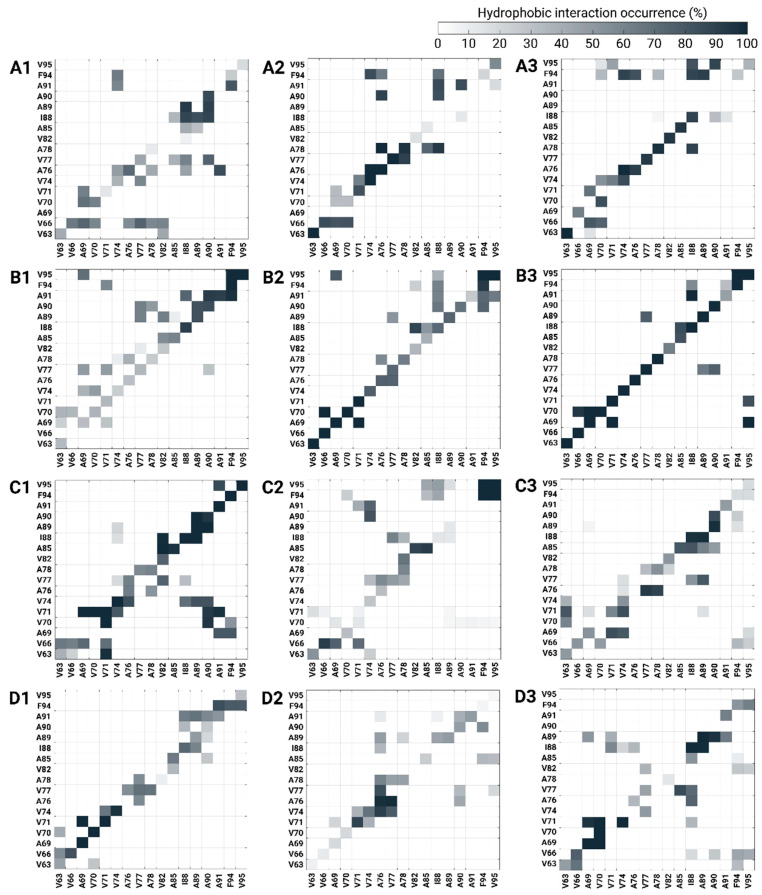
Hydrophobic interaction occurrence NAC contact maps between two Cu^2+^-bound AS monomers within the dimer models at low copper concentrations (models A1–A3, B1–B3, C1–C3, D1–D3).

To interpret these observations, we computed contact maps of the hydrophobic interactions between monomers within the polymorphic AS dimers at low copper concentrations (Figure 6). In models A3, B3, C3, and D3, where copper ions bind to the C-terminal domain, the hydrophobic interactions between monomers within the dimers were more pronounced along the diagonal contacts. In these models, there is less competition between other interactions at non-diagonal contacts. In cases where copper ions bound to His50 in models A2, B2, and D2, strong short or long segments of diagonal hydrophobic interactions in the NAC domain were presented with less competing non-diagonal hydrophobic interactions in the contact maps. Finally, the strong diagonal hydrophobic interactions along the NAC domain of models C1 and C3 revealed a relatively low conformational energy when compared with that of model C2.

In summary, all polymorphic AS dimers that bind copper ions at the C-termini at low copper concentrations are more populated and preferred because of the strong hydrophobic contacts between the NAC domains within the dimers. All polymorphic AS dimers, excluding model C, by which copper ions bind to His50, are also preferred because of the hydrophobic contacts between the NAC domains. Finally, among all polymorphic AS dimers, only model C exhibited a preference to bind copper ions in the N-terminal domains at low copper concentrations.

### 3.4. Copper-Binding Site at the C-Terminus at High Concentrations Is Preferred Due to Conservation of Hydrophobic Contacts in the NAC Domain

The copper concentration is a crucial condition that affects AS aggregation. The exposure of AS monomers and oligomers to high copper concentrations induces AS aggregation [15]. However, the effect of high copper concentrations on the preference of copper-binding sites in polymorphic AS oligomers has not been reported.

**Figure 7:**
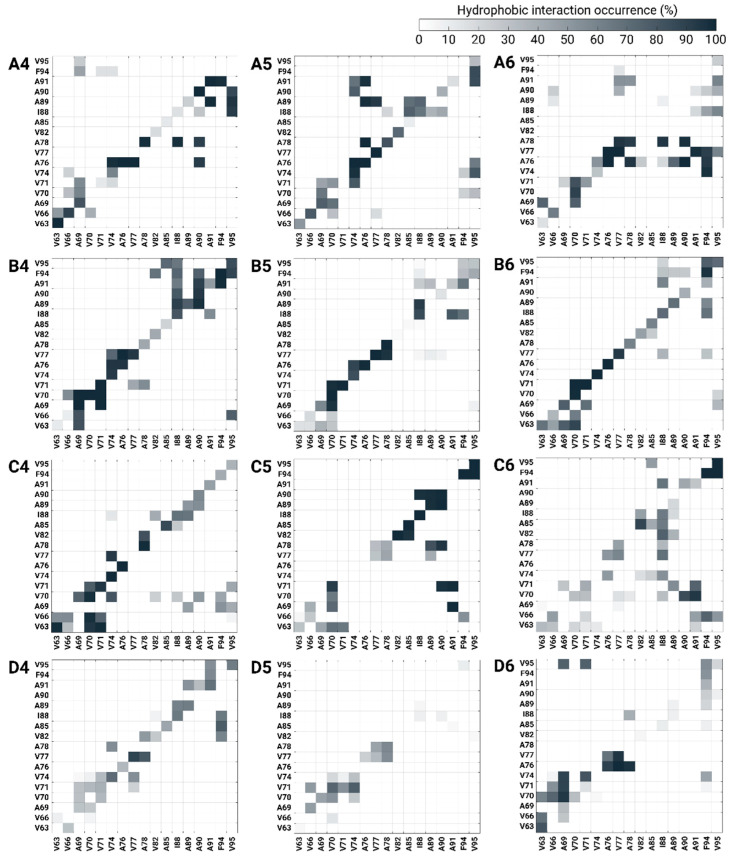
Hydrophobic interaction occurrence NAC contact maps between two Cu^2+^-bound AS monomers within the dimer models at high copper concentrations (models A4–A6, B4–B6, C4–C6, D4–D6).

Herein, three copper-binding sites were examined in four distinct polymorphic AS dimers. The conformational energy and population of each polymorphic copper-AS dimer complex were computed at high copper concentrations (Figure 5b). Interestingly, at high copper concentrations, all polymorphic copper-AS dimer complexes in which copper ions were bound to the C-termini (models A6, B6, C6, and D6) presented lower (or the lowest) conformational energies. These polymorphic copper-AS dimer complex models exhibited strong hydrophobic contacts between the NAC domains of the monomers compared to other polymorphic copper-AS dimer complexes, in which copper ions were bound to N-termini or His50 at high copper concentrations (Figure 7). Moreover, fewer and weak competitive hydrophobic interactions (non-diagonal interactions) were uncovered in polymorphic copper-AS dimer complexes by which copper ions were bound to the C-terminus.

### 3.5. The Preference of Copper-Binding Sites Depends on Copper Concentrations and on the Polymorphic AS Dimers

Polymorphism in amyloids has been well-recognized for more than 60 years, specifically for amyloid fibrils [63]. AS fibrils also presented polymorphic states [26,27,28]. The expression of amyloid fibril polymorphs from the pathways of their production from the original oligomers indicate that amyloid oligomers are also polymorphic. Herein, we investigated four distinct polymorphic AS dimers. Three different copper-binding sites were examined for each polymorphic AS dimer at low and high copper concentrations. It is interesting to study the preference of the copper-binding site for each polymorphic dimer both at low and high copper concentrations.

At low and high copper concentrations, the copper-binding sites that are preferred in the copper-AS dimer complex model C include the N- and C-termini (models C1, C3, C4, and C6). At low and high copper concentrations, the copper-binding sites that are preferred in the copper-AS dimer complex model D include His50 and the C-terminus (models D2, D3, D5, and D6). At low copper concentrations, the copper-binding sites that are preferred in the copper-AS dimer complex models A and B include His50 and the C-terminus (models A2, A3, B2, and B3), and, at high copper concentrations, these include the N- and C-termini for model A (models A4 and A6), and the C-terminal for model B (model B6).

Thus, we suggest that the preference of the specific copper-binding site in the AS dimer strongly depends on the polymorph and copper concentrations. This phenomenon, thus, illustrates a complexity when discussing polymorphism in AS oligomers, particularly when investigating therapeutics to inhibit the primary nucleation of AS.

### 3.6. The Original Structure of AS Dimer Affects the Flexibility or Rigidity of Copper-AS Dimer Complexes

**Figure 8:**
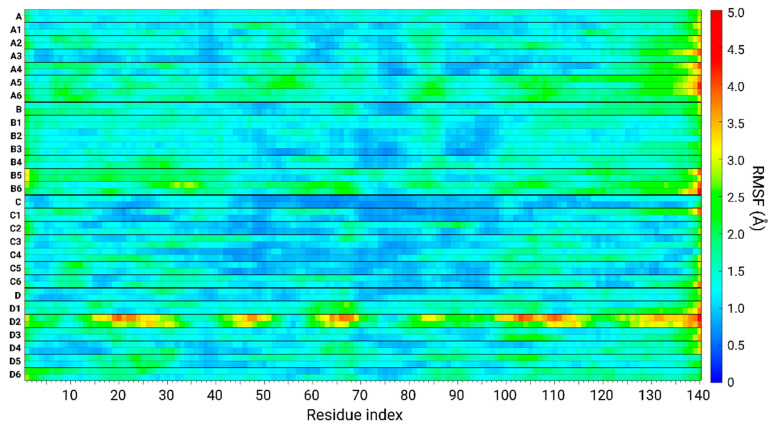
Root-mean-square fluctuation (RMSF) values of along the sequence of AS in Cu^2+^-free polymorphic AS_1–140_ dimers (models A, B, C, and D) [5] and in Cu^2+^-bound polymorphic AS_1–140_ dimers (models A1–A6, B1–B6, C1–C6, and D1–D6).

The structural stability, flexibility, rigidity, and dynamics of different domains within the AS sequence of the studied copper-AS dimer complexes can be compiled from RMSF analyses. The rigidity and flexibility of the residues along the sequences of the AS dimer models are displayed in Figure 8. The models of AS dimers include AS dimers in the absence of copper ions—models A, B, C, and D—and AS dimers that bound copper ions at distinct binding sites for each model.

Model C and models C1–C6 showed a relative rigidity along the sequence of AS. Among all the models studied here, these models present the highest rigidity. Specifically, rigidity was mainly observed along the NAC domain of these models. Interestingly, among all the models studied, the RMSDs of these models demonstrated the lowest values (Figures S5–S8). In these models, the NAC domains of the dimers were packed and buried in the core of the structure (Figure 2), and the monomers demonstrated hydrophobic interactions along the NAC domain (Figure 5 and Figure 6). These models are derived from the AS fibril that exhibited a buried NAC domain [36].

Models A, B, and models A1–A6 and models B1–B6 revealed less rigidity than models C and models C1–C6. The fluctuations and flexibility of the residues of these models are mainly represented at the N- and C-termini (Figure 8). These dimers were derived from models A and B, fibrils that were solved by experimental techniques and investigated by computational studies [19]. The NAC domains in these fibrils are less buried in the core, and the N- and C-termini are more exposed to the solution; thus, the dimers fluctuated more than the dimers derived from model C. Specifically, the dimers derived from model B had higher RMSD values than the dimers derived from model A (Figures S5–S8). Moreover, the dimers derived from model B are more exposed than those derived from model A; hence, they are more solvated in water (Figure S11).

Finally, AS dimer models that are derived from model D and bind copper ions (models D1–D6) demonstrated the most flexible domains over all the AS sequence: the NAC domain and N- and C-termini (Figure 8). The NAC domains in model D were relatively more exposed and less packed in the core of the fibrillary cross-β structure. Moreover, the N-termini and, particularly, the C-termini of model D were also less packed and formed less contacts with the core of the cross-β fibrillary structure. Therefore, the AS dimer models derived from model D fluctuate more than other AS dimer models. The highest fluctuations were observed remarkably in models D2 and D5. In these dimers, copper ions were bound to His50 residues at low and high copper concentrations. High fluctuations are also recognized in the case of the AS dimer derived from model D, which binds copper ions at the C-termini at high copper concentrations (model D6).

In summary, the rigidity and flexibility of polymorphic copper-bound AS dimers depend on their original fibrillar morphology. There may be fibrils in which the structural properties of flexibility or rigidity depend on the copper-binding sites and copper concentrations. Evidently, the polymorphism of copper-AS dimers increases the complexity of using experimental techniques to make observations. Herein, computational methods allowed us to provide insights into the molecular mechanisms of copper binding to polymorphic AS dimers at the atomic resolution.

## 4. Conclusions

The primary nucleation of AS and the total aggregation process of AS are affected by the presence of copper ions [9,11,12,13,14,15]. It is well-known that AS fibrils and oligomers are polymorphic [26,27,28,31,32]. The effect of copper ions on polymorphic AS fibrils has been investigated at the molecular level [19]. To precisely investigate whether copper ions accelerate or inhibit the primary nucleation of polymorphic AS early-stage oligomers, we examined two factors that affect this process: (1) the copper concentrations, and (2) the copper-binding sites.

**Figure 9:**
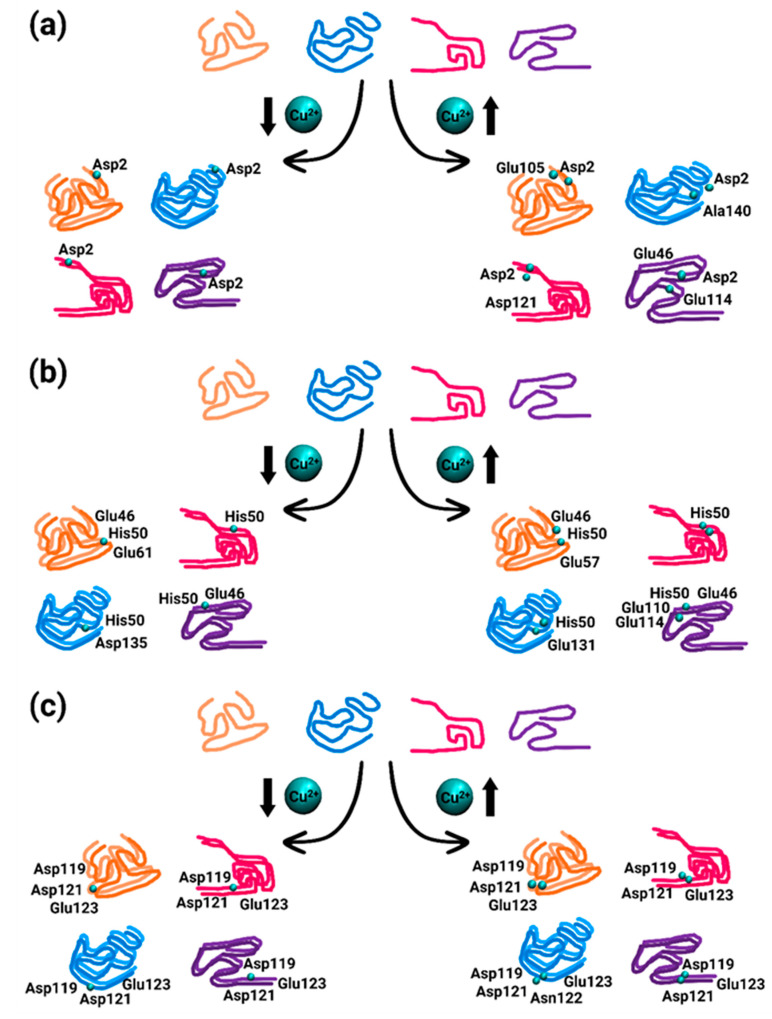
Proposed mechanisms for Cu^2+^-binding sites in polymorphic AS dimers models A (orange), model B (pink), model C (blue), and model D (purple): The Cu^2+^ concentrations affect the specific Cu^2+^-binding site: (**a**) At low Cu^2+^ concentration, the binding is preferred by Asp2, whereas, at high Cu^2+^ concentration, additional residues participate in the Cu^2+^-binding. (**b**) Both at low and high Cu^2+^ concentrations, the His50 binds to Cu^2+^ and to further residues from both N- and C-termini domains. (**c**) Both at low and high Cu^2+^ concentrations, a common Cu^2+^-binding site appears at the C-termini: Asp119, Asp121, and Glu123 (excluding in model C).

Our extensive MD simulations have led to novel and diverse conclusions. These conclusions led us to propose molecular mechanism pathways by which copper concentrations affect the binding sites in distinct polymorphic AS dimers (Figure 9). First, copper ions that bind to the N-terminus of polymorphic AS dimers at high copper concentrations allowed for further residues aside from Asp2 to complete the co-ordination mode. Second, at both high and low copper concentrations, copper ions bind to His50 in all polymorphic AS dimers, and further Glu or Asp residues complete the metal co-ordination mode, excluding polymorphic model B, in which only His50 contributes to the metal co-ordination mode. Third, at both high and low copper concentrations, all polymorphic AS dimers bound copper ions at the C-termini: residues Asp119, Asp121, and Glu123 (and not to Asn122). Fourth, the AS primary nucleation process strongly depends on the copper concentration and the specific copper-binding site. Fifth, at low copper concentrations, the preference of copper-binding sites depends on the hydrophobic contacts between the NAC domains within the AS dimer models. Sixth, at high copper concentrations, the C-terminal binding site is preferred because of the strong hydrophobic contacts between the NAC domains within the AS dimers. Seventh, overall, the preference of copper-binding sites changes from one AS polymorph dimer to other polymorph dimers and depends on the copper concentration.

Our extensive simulations of polymorphic AS dimers provide the first insight into the distinct molecular mechanism pathways for primary nucleation that depend on two factors: copper concentration and specific copper-binding sites. Our study illustrates the complexity of the diverse effects of copper ions on AS primary nucleation. Yet, the knowledge gained from the current study will allow future in vitro and in vivo studies to investigate potential inhibitors that can eliminate the primary nucleation of polymorphic AS dimers in the presence of copper ions.

## Figures and Tables

**Table 1:** Classification of the 24 Cu^2+^-bound AS dimers, based on four polymorphic fibrils: A (Ref. [34]), B (Ref. [35]), C (Ref. [36]), and D (Ref. [36]). The symbols A–D indicate four polymorphic dimers that are based on polymorphic AS fibrils. Models that consist of numbers 1 and 4 designate the binding site at the N-terminal domain, involving Met1, Asp2, and Met5. Models that consist of numbers 2 and 5 mark the binding site of His50, and models that consist of numbers 3 and 6 identify the binding site at the C-terminal domain, involving Asp119, Glu121, Asn122, and Glu123. Finally, the numbers 1, 2, and 3 classify low copper concentrations and the numbers 4, 5, and 6 classify high copper concentrations.

		Cu^2+^ Binding Site		
Polymorph-Based Fibril	Cu^2+^:AS Ratio	Met1, Asp2, Met5	His50	Asp119, Asp121, Asn122, Glu123
A	1:2	A1	A2	A3
	1:1	A4	A5	A6
B	1:2	B1	B2	B3
	1:1	B4	B5	B6
C	1:2	C1	C2	C3
	1:1	C4	C5	C6
D	1:2	D1	D2	D3
	1:1	D4	D5	D6

## Data Availability

The data presented in this study are available on request from the corresponding author.

## Supplementary Materials

The following supporting information can be downloaded at: https://www.mdpi.com/article/10.3390/biom14060627/s1, Figure S1: Initial constructed models of Cu^2+^-bound AS_1–140_ dimers based on a computational fibril model A; Figure S2: Initial constructed models of Cu^2+^-bound AS_1–140_ dimers based on ssNMR fibril model B; Figure S3: Initial constructed models of Cu^2+^-bound AS_1–140_ dimers based on cryo-EM fibril model C; Figure S4: Initial constructed models of Cu^2+^-bound AS_1–140_ dimers based on cryo-EM fibril model D; Figure S5: RMSD values for low Cu^2+^ concentration and Cu^2+^-free AS_1–140_ dimer models; Figure S6: RMSD values for high Cu^2+^ concentration and Cu^2+^-free AS_1–140_ dimer models; Figure S7: RMSD values of NAC domain for low Cu^2+^ concentration and Cu^2+^-free AS_1–140_ dimer models; –S8: RMSD values of NAC domain for high Cu^2+^ concentration and Cu^2+^-free AS1–140 dimer models; Figure S9: Hydrogen bond analyses of all models; Figure S10: Average number of water molecules around amino acids of Cu^2+^-bound AS_1–140_ dimers; Figure S11: Average number of water molecules along the simulations of Cu^2+^-bound AS_1–140_ dimers.
