# Supplementary material for: Primary Nucleation of Polymorphic α-Synuclein Dimers Depends on Copper Concentrations and Definite Copper-Binding Site
Source: Biomolecules. 2024 May 26;14(6):627. doi: 10.3390/biom14060627 (PMC11201572; doi:10.3390/biom14060627)
Supplement: Supplementary file 1 [file biomolecules-14-00627-s001.zip › biomolecules-3016078-supplementary.pdf]

Supporting Information for

"Primary nucleation of polymorphic α-synuclein dimers depends on copper concentrations and definite copper binding site"

Carmia Blacher,[1,2,3,‡] Karina Abramov-Harpaz [1,2,3,‡] and Yifat Miller[1,2,3*]

[1]Department of Chemistry, Ben-Gurion University of the Negev, P.O. Box 653, Be'er Sheva 8410501, Israel

[2] Ilse Katz Institute for Nanoscale Science and Technology, Ben-Gurion University of the Negev, Beér-Sheva 8410501, Israel

[3] The School of Brain Sciences and Cognition, Ben-Gurion University of the Negev, Beér-Sheva 8410501, Israel

Corresponding author: Yifat Miller

ymiller@bgu.ac.il

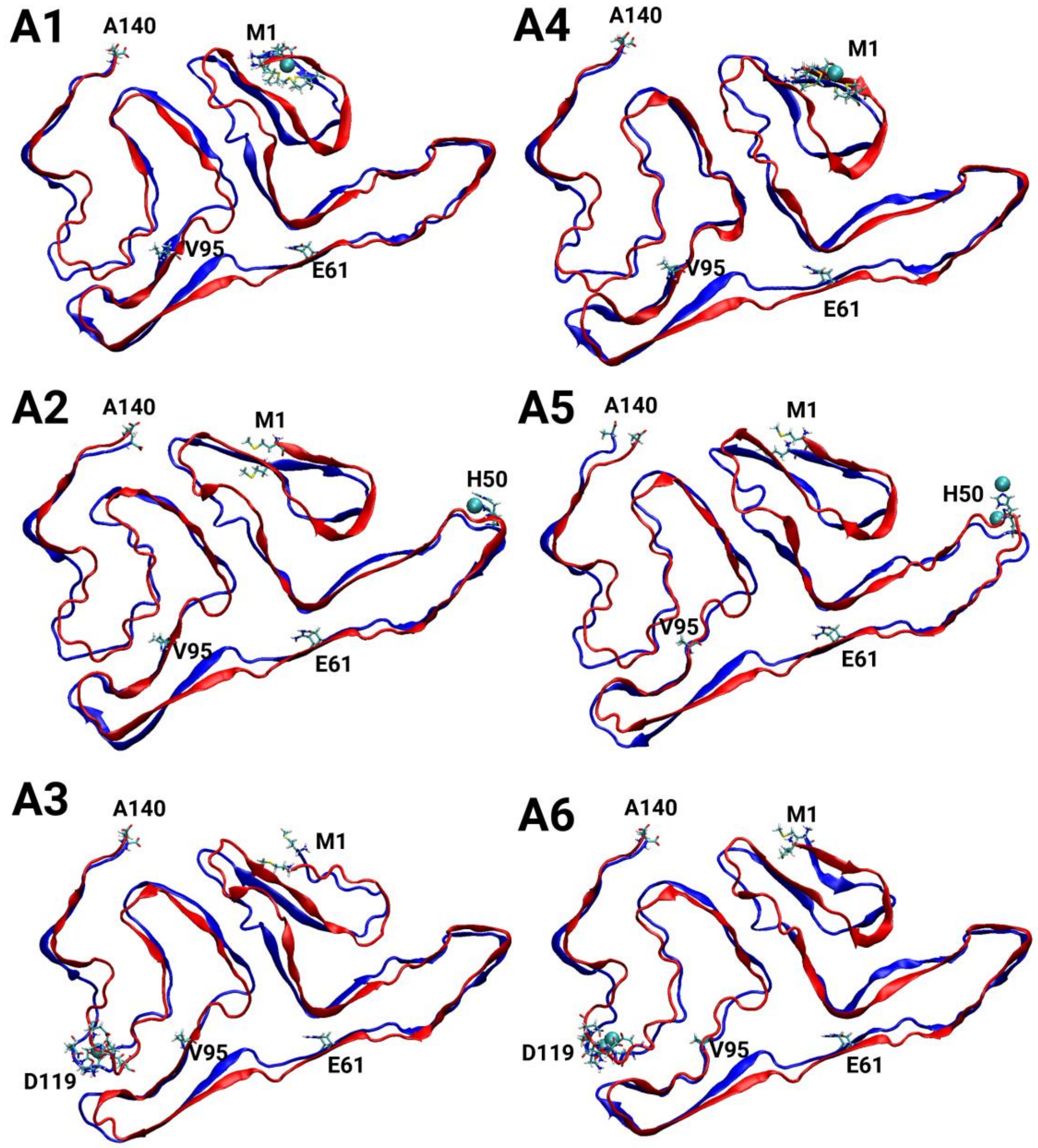

Figure S1: Initial constructed models of Cu^{2+}-bound $AS_{1\text{-}140}$ dimers, based on a computational fibril model:[1] In models A1-A3 the Cu^{2+} ions are bound in Cu^{2+}:AS ratio of 1:2 (low concentration), and in models A4-A6 the Cu^{2+} ions are bound in Cu^{2+}:AS ratio of 1:1 (high concentration). In models A1 and A4, the Cu^{2+} ions are bound to Met1, Asp2, and Met5. In models A2 and A5, the Cu^{2+} ions are bound to His50. In models A3 and A6, the Cu^{2+} ions are bound to Asp119, Glu121, Asn122, and Glu123.

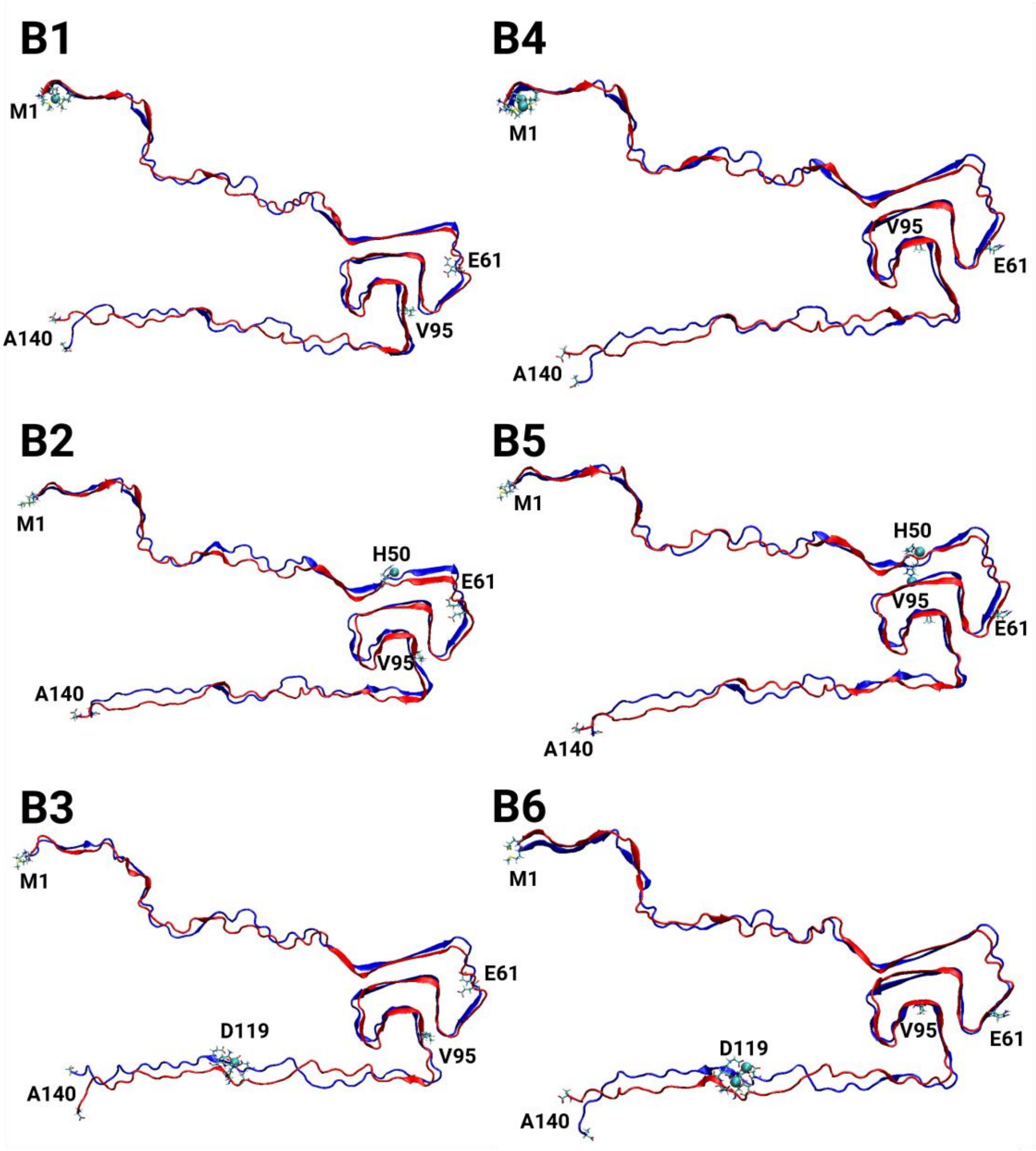

Figure S2: Initial constructed models of Cu^{2+}-bound AS_{1-140} dimers, based on a ssNMR fibril model:[2] In models B1-B3 the Cu^{2+} ions are bound in Cu^{2+}:AS ratio of 1:2 (low concentration) and in models B4-B6 the Cu^{2+} ions are bound in Cu^{2+}:AS ratio of 1:1 (high concentration). In models B1 and B4, the Cu^{2+} ions are bound to Met1, Asp2, and Met5. In models B2 and B5, the Cu^{2+} ions are bound to His50. In models B3 and B6, the Cu^{2+} ions are bound to Asp119, Glu121, Asn122, and Glu123.

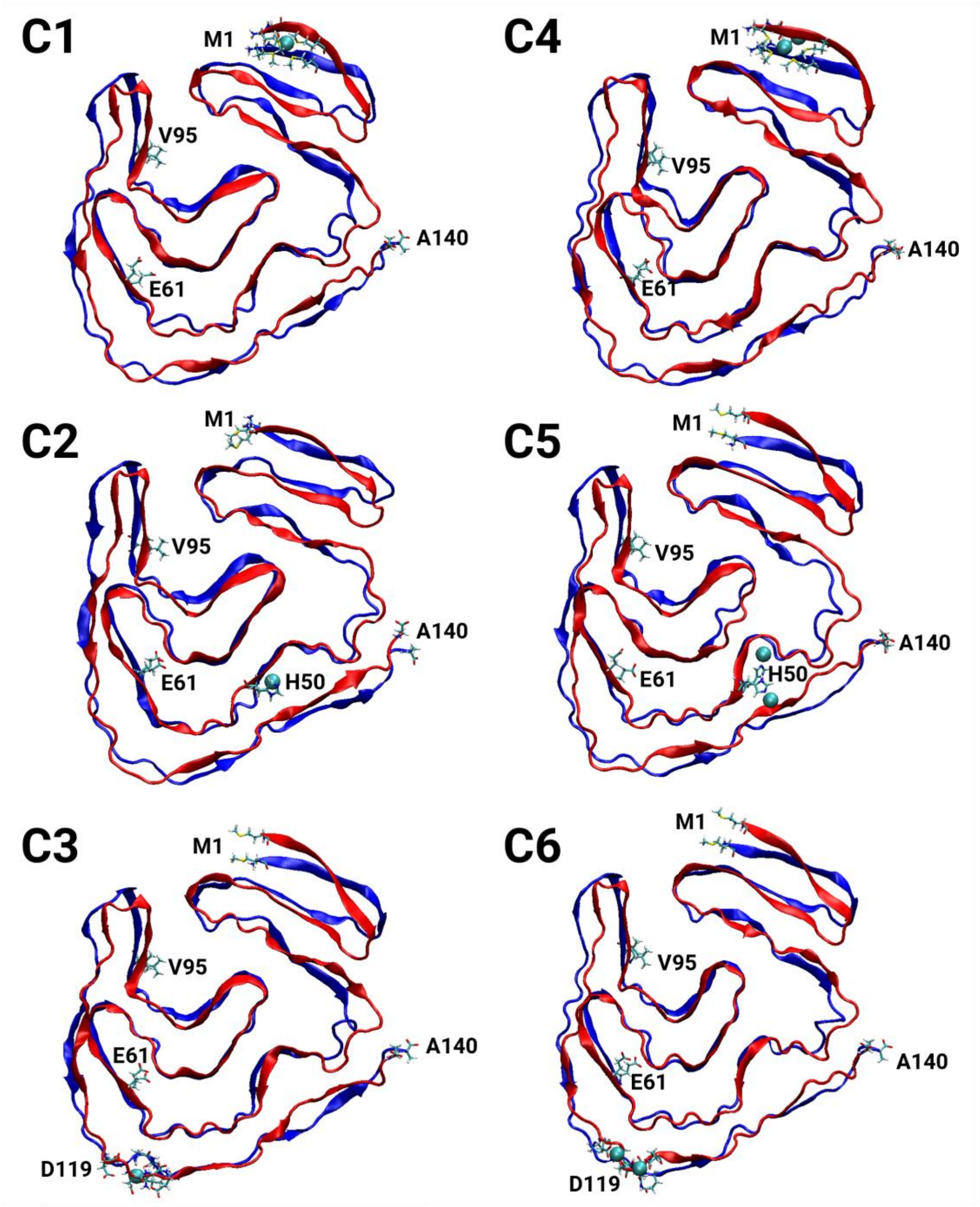

Figure S3: Initial constructed models of Cu^{2+}-bound AS_{1-140} dimers, based on cryo-EM fibril model:[3] In models C1-C3 the Cu^{2+} ions are bound in Cu^{2+}:AS ratio of 1:2 (low concentration) and in models C4-C6 the Cu^{2+} ions are bound in Cu^{2+}:AS ratio of 1:1 (high concentration). In models C1 and C4, the Cu^{2+} ions are bound to Met1, Asp2, and Met5. In models AC and C5, the Cu^{2+} ions are bound to His50. In models C3 and C6, the Cu^{2+} ions are bound to Asp119, Glu121, Asn122, and Glu123.

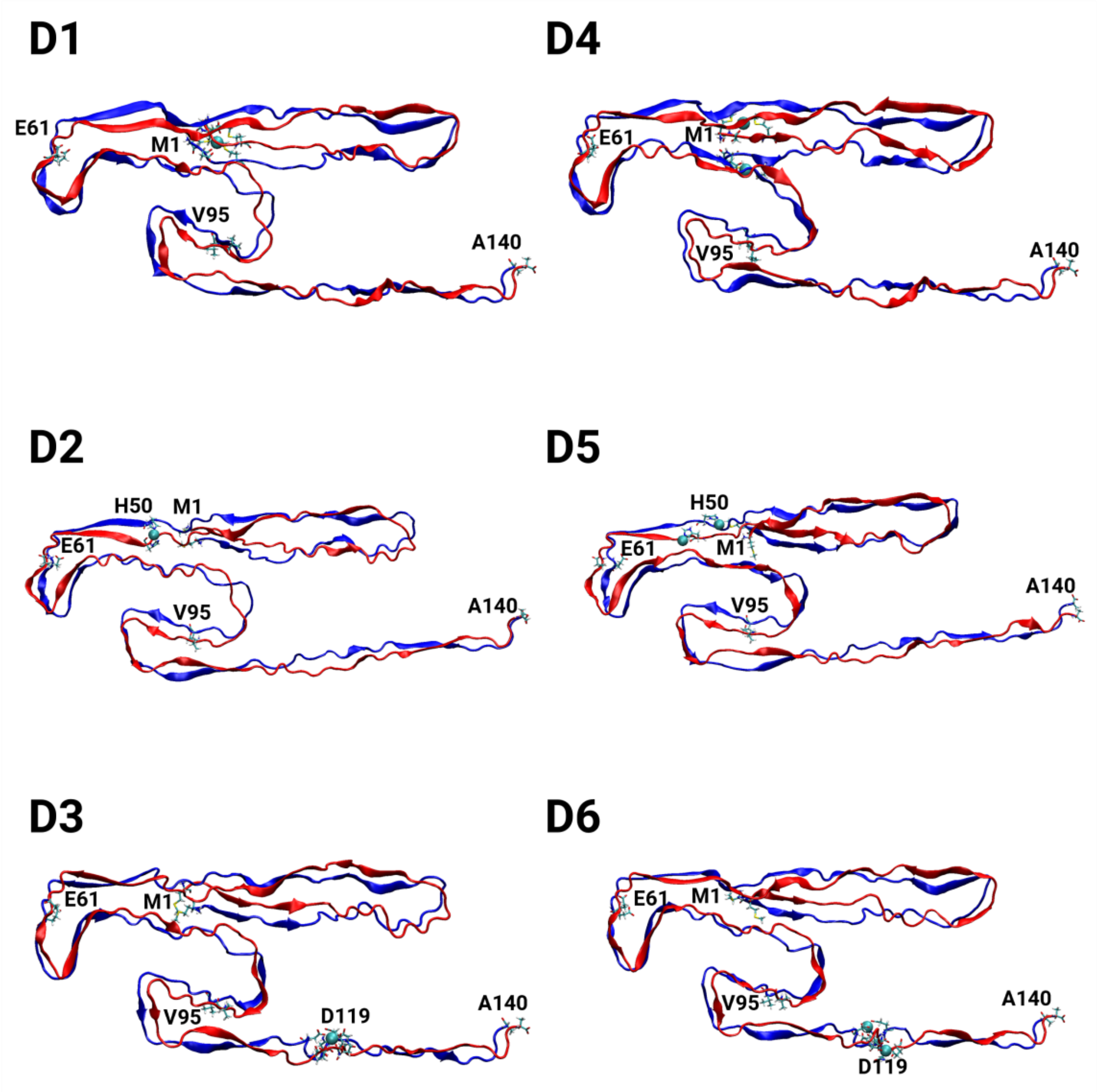

Figure S4: Initial constructed models of Cu^{2+}-bound AS_{1-140} dimers, based on cryo-EM fibril model:[3] In models D1-D3 the Cu^{2+} ions are bound in Cu^{2+}:AS ratio of 1:2 (low concentration) and in models D4-D6 the Cu^{2+} ions are bound in Cu^{2+}:AS ratio of 1:1 (high concentration). In models D1 and D4, the Cu^{2+} ions are bound to Met1, Asp2, and Met5. In models D2 and D5, the Cu^{2+} ions are bound to His50. In models D3 and D6, the Cu^{2+} ions are bound to Asp119, Glu121, Asn122, and Glu123.

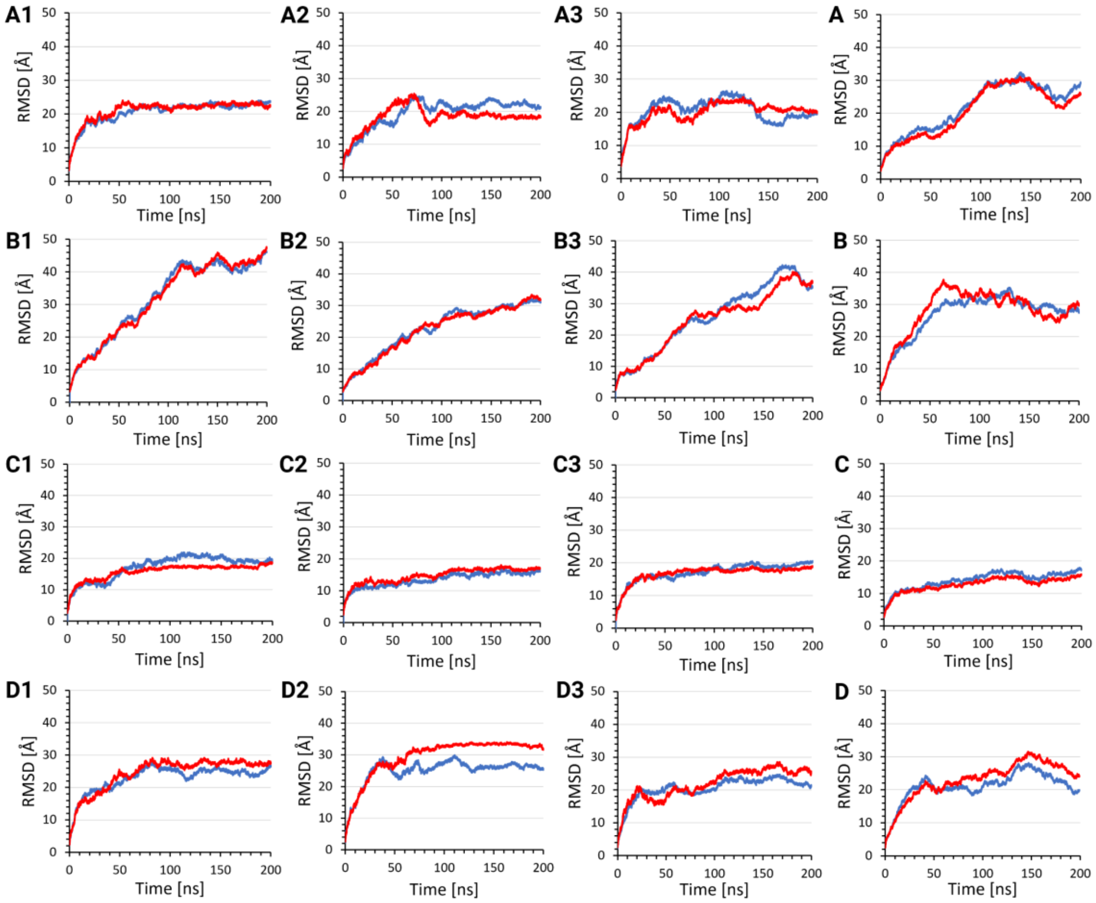

Figure S5: RMSD values of AS_{1-140} along the MD simulations for the Cu^{2+}-bound AS_{1-140} dimers (models A1-A3, B1-B3, C1-C3 and D1-D3) and Cu^{2+}-free AS_{1-140} dimers (models A, B, C and D).

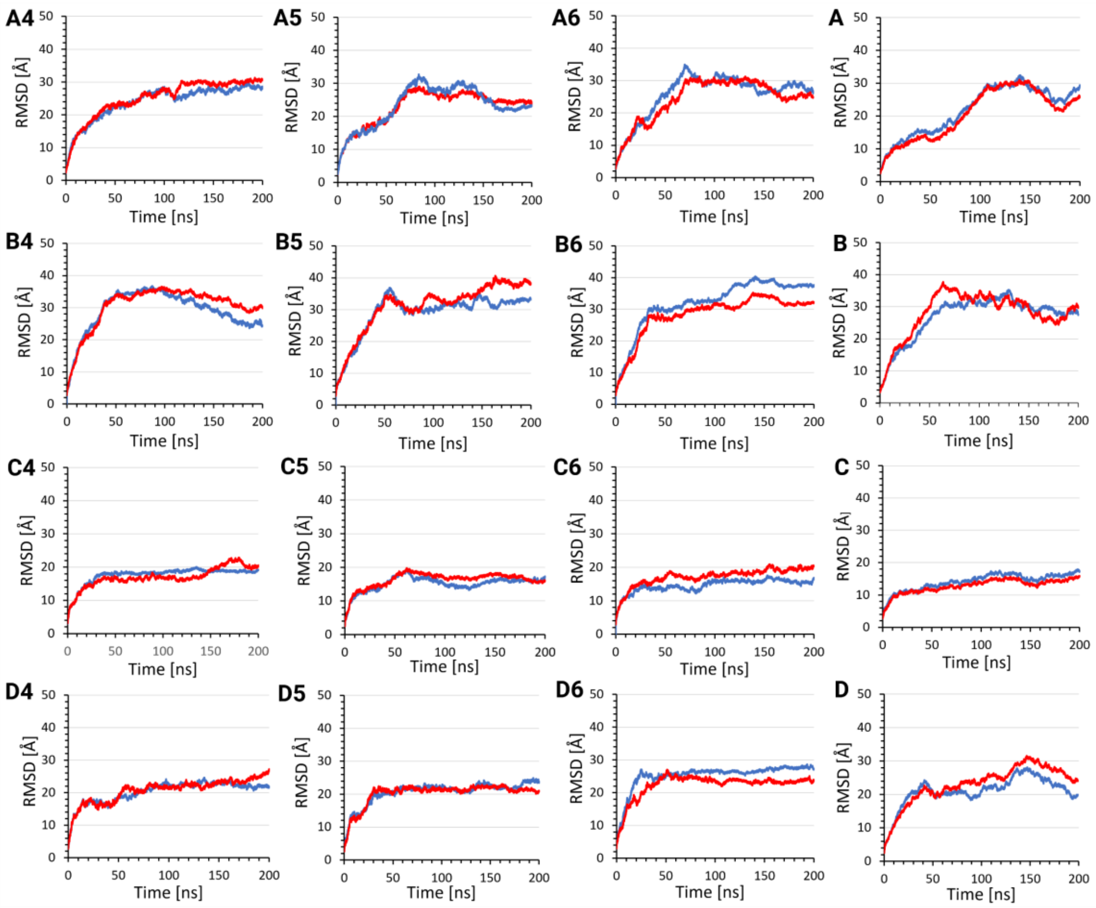

Figure S6: RMSD values of AS_{1-140} along the MD simulations for the Cu^{2+}-bound AS_{1-140} dimers (models A4-A6, B4-B6, C4-C6 and D4-D6) and Cu^{2+}-free AS_{1-140} dimers (models A, B, C and D).

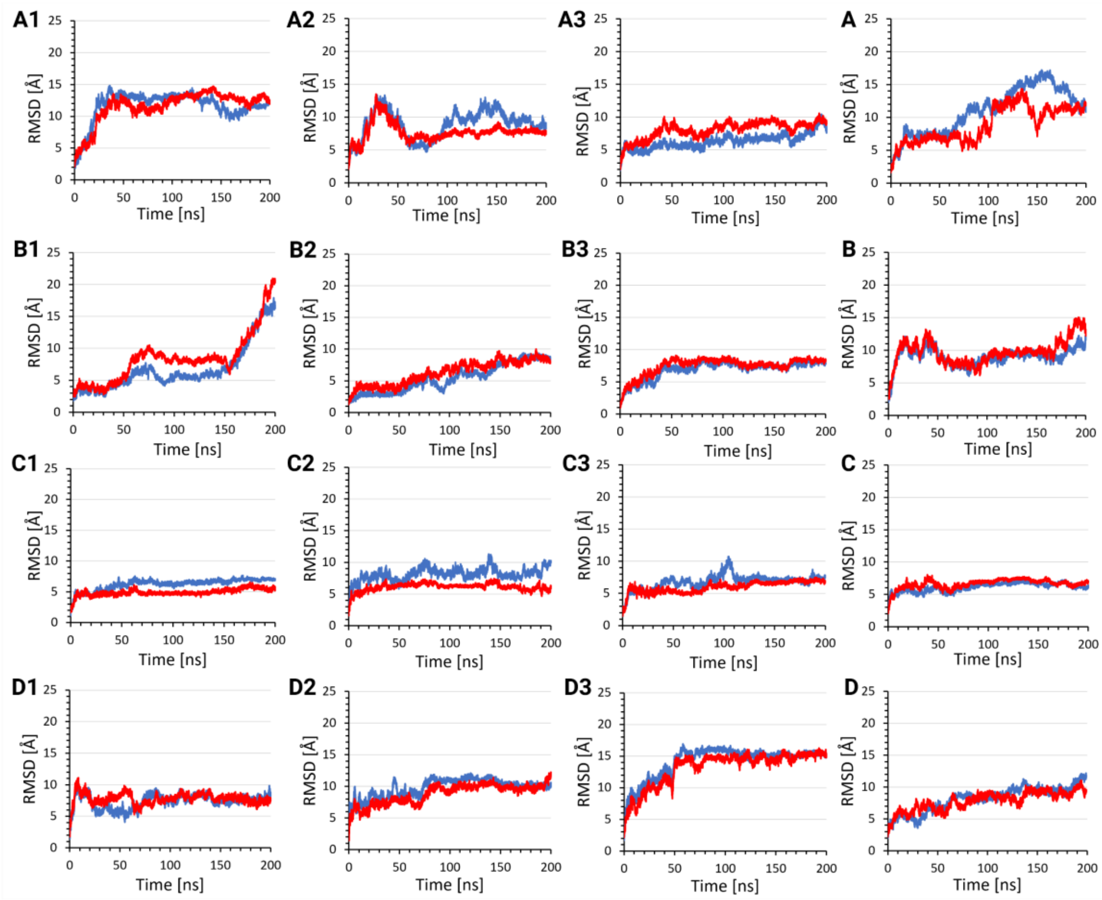

Figure S7: RMSD values of NAC along the MD simulations for the Cu^{2+}-bound AS_{1-140} dimers (models A1-A3, B1-B3, C1-C3 and D1-D3) and Cu^{2+}-free AS_{1-140} dimers (models A, B, C and D).

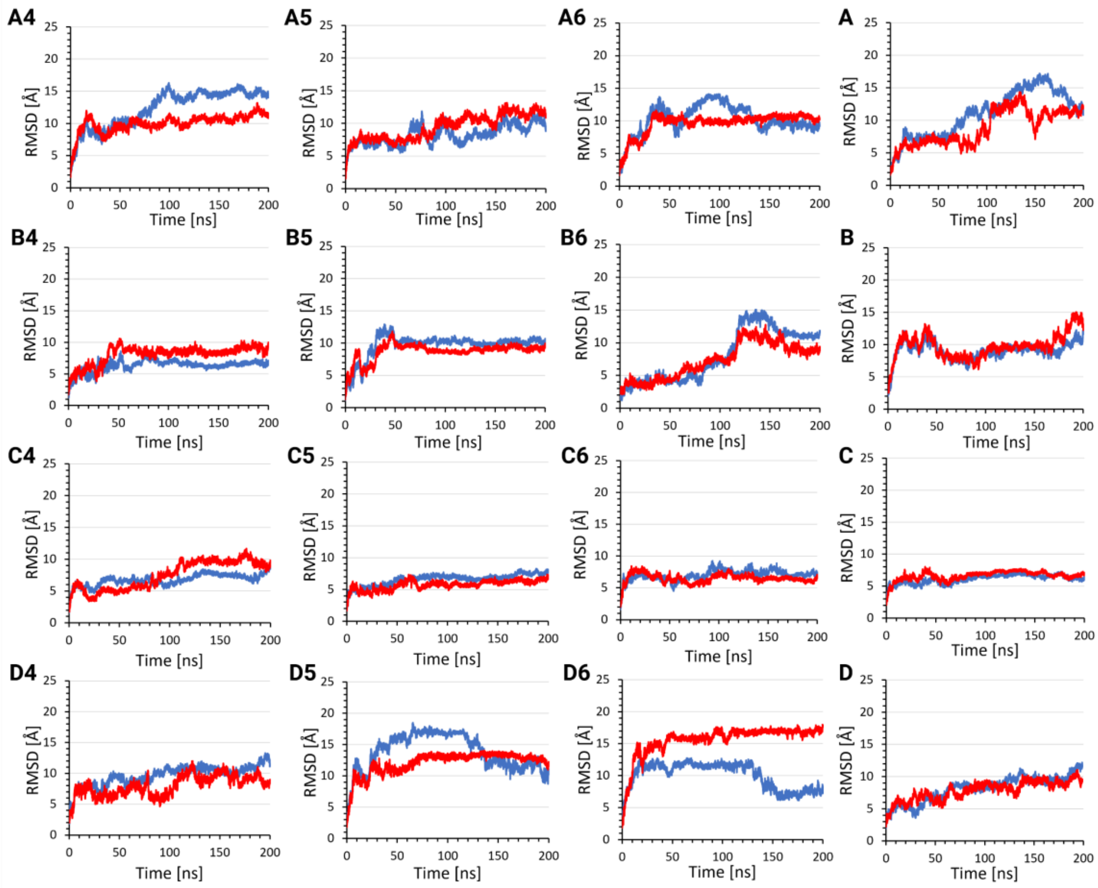

Figure S8: RMSD values of NAC along the MD simulations for the Cu^{2+}-bound AS_{1-140} dimers (models A4-A6, B4-B6, C4-C6 and D4-D6) and Cu^{2+}-free AS_{1-140} dimers (models A, B, C and D).

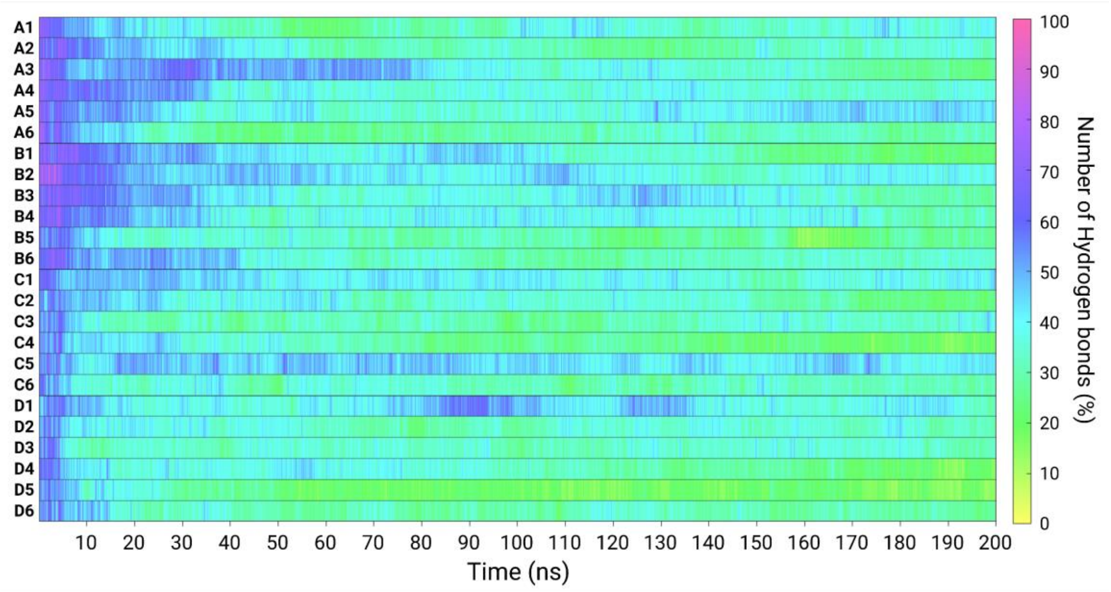

Figure S9: The total number of hydrogen bonds between the Cu^{2+}-bound AS dimers was normalized to percentages for all models along the MD simulations.

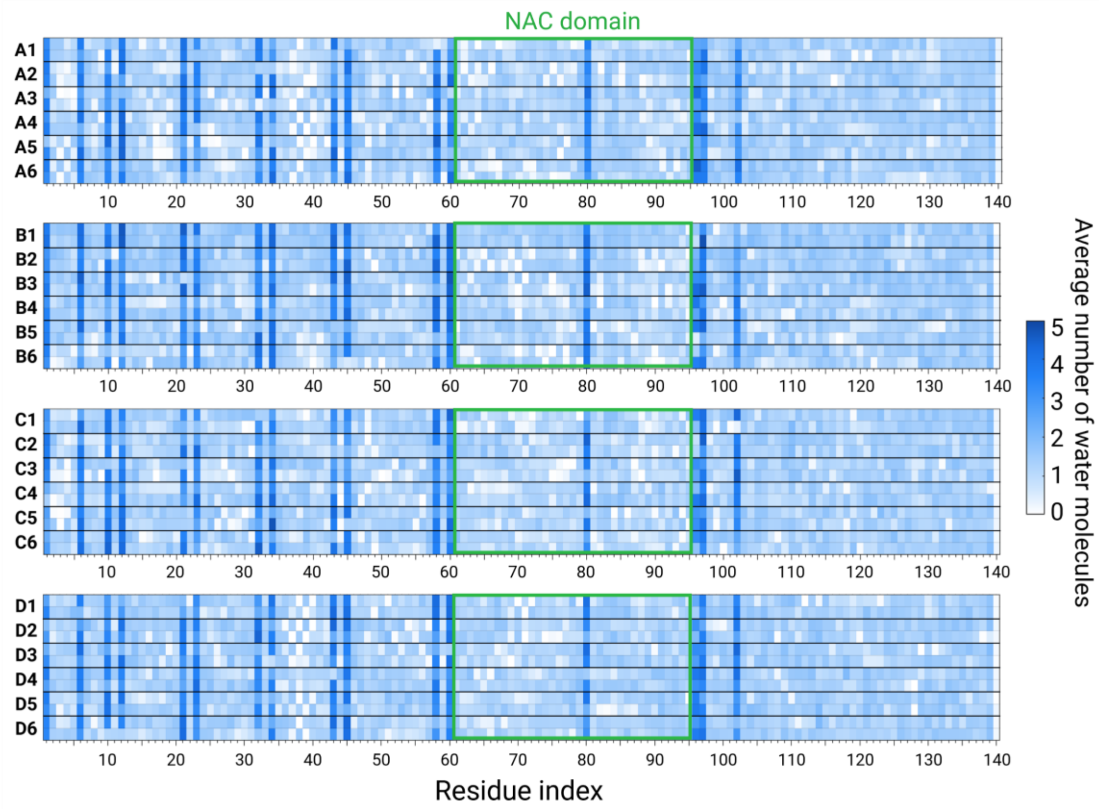

Figure S10: Average number of water molecules around each amino acid in the sequence of each AS monomer within the Cu^{2+}-bound AS_{1-140} dimer models. The NAC domain sequence is indicated in green rectangles.

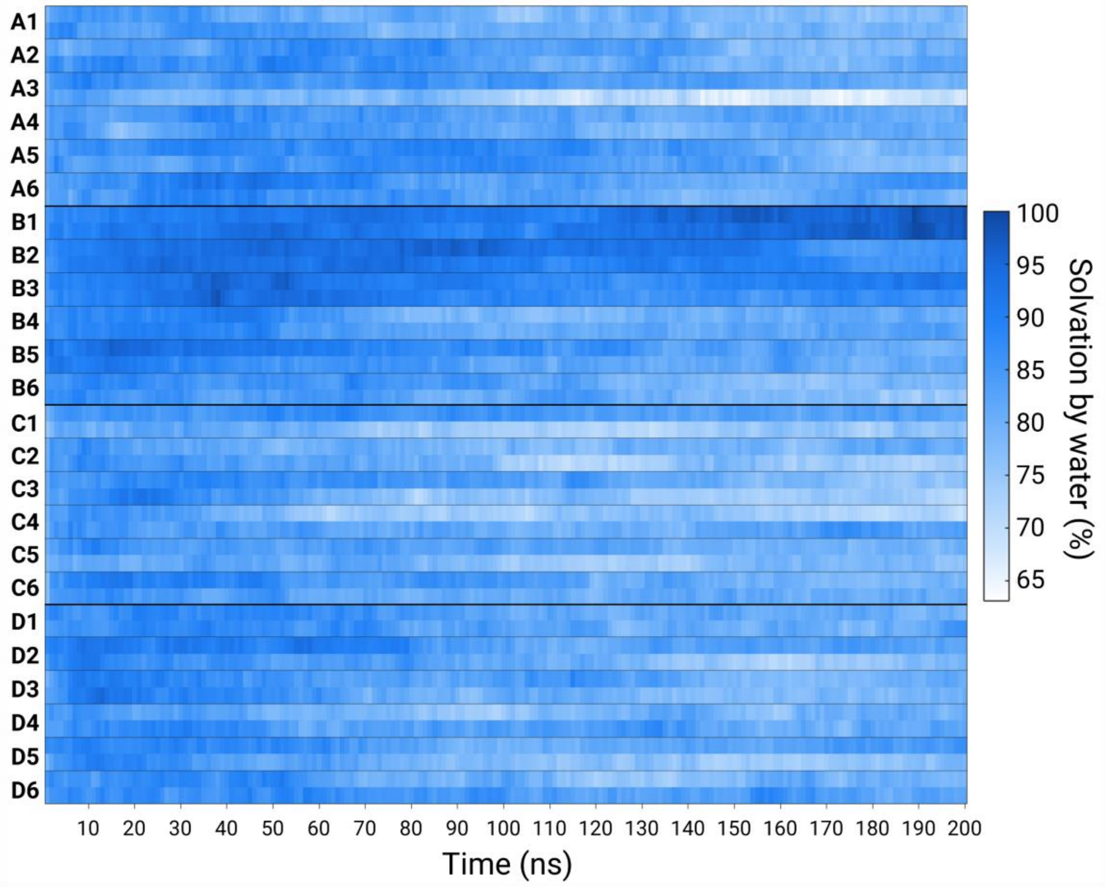

Figure S11: Average number of water molecules around each AS monomer within the Cu^{2+}-bound AS_{1-140} dimer models along the MD simulations normalized to percentages.

References

1. Bloch, D. N.; Miller, Y., Study of Molecular Mechanisms of alpha-Synuclein Assembly: Insight into a Cross-beta Structure in the N-Termini of New alpha-Synuclein Fibrils. *ACS Omega* 2017, 2, 3363-3370.
2. Tuttle, M. D.; Comellas, G.; Nieuwkoop, A. J.; Covell, D. J.; Berthold, D. A.; Kloepper, K. D.; Courtney, J. M.; Kim, J. K.; Barclay, A. M.; Kendall, A.; Wan, W.; Stubbs, G.; Schwieters, C. D.; Lee, V. M.; George, J. M.; Rienstra, C. M., Solid-state NMR structure of a pathogenic fibril of full-length human alpha-synuclein. *Nat Struct Mol Biol* 2016, 23, 409-15.
3. Li, B.; Ge, P.; Murray, K. A.; Sheth, P.; Zhang, M.; Nair, G.; Sawaya, M. R.; Shin, W. S.; Boyer, D. R.; Ye, S.; Eisenberg, D. S.; Zhou, Z. H.; Jiang, L., Cryo-EM of full-length alpha-synuclein reveals fibril polymorphs with a common structural kernel. *Nat Commun* 2018, 9, 3609.
